# Chiropractic management of post spinal cord stimulator spine pain: a case report

**DOI:** 10.1186/s12998-017-0136-0

**Published:** 2017-02-06

**Authors:** Rachel M. Perrucci, Christopher M. Coulis

**Affiliations:** 1 Michael E. DeBakey VA Medical Center, Rehabilitation Care Line, 580/RECL 117, 2002 Holcombe Boulevard, Houston, TX 77030 USA; 2 VA Connecticut Healthcare System, Physical Medicine and Rehabilitation, 950 Campbell Ave, West Haven, CT 06516 USA

**Keywords:** Chiropractic, Spinal cord stimulator, Failed back surgery syndrome, Post-surgical spine pain

## Abstract

**Background:**

A brief overview of failed back surgery syndrome, with emphasis on low back pain status post spinal cord stimulation, and post-surgical spinal manipulation is presented.

**Case Presentation:**

Four cases of patients within the VA Connecticut Health Care System presenting between July 2014 and July 2015 reporting low back pain after surgical insertion of spinal cord stimulators are discussed. This study describes the outcomes experienced by four patients with low back pain status post implanted spinal cord stimulators receiving manual therapy in the form of lumbar spine manipulation or mobilization.

**Conclusion:**

All four patients denied adverse effects to spinal manipulation/mobilization and onset of new symptoms after treatment; two patients reported durable reduction in low back pain with increased tolerance to walking, standing, or lying down, one reported temporary relief of low back pain, and one reported no change in symptoms. Further investigation is needed to determine the benefit of spinal manipulation in patients with implanted spinal cord stimulators, but this study has shown the absence of adverse effects from manipulation or mobilization treatment, in regards to SCS.

## Background

**Fig. 1:**
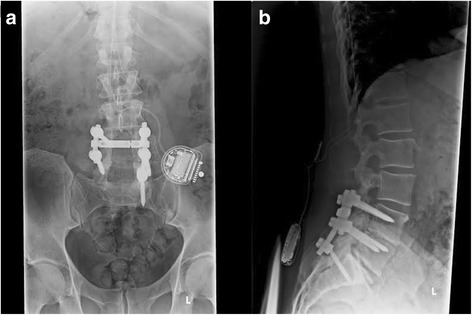
Radiographs of a lumbar spine status post lumbar decompression, fusion, and implantation of spinal cord stimulator. Visualized is a series of two radiographs, including an anterioposterior view (**a**), and a lateral view (**b**), of a post-surgical lumbar spine with pedicle screw and rod fixation at L4-S1 levels, and spinal cord stimulator lead wires entering the spinal column at L1-2 with a pulse generator over the left iliac crest

Spinal cord stimulation (SCS) has been used since 1967 for the treatment of chronic pain [1–5]. In the United States, SCS is approved by the Food and Drug Administration for chronic trunk and limb pain, intractable low back pain, leg pain, and failed back surgery syndrome (FBSS) [3]. Of the previously mentioned conditions, the current most common indicator for SCS is FBSS [1–3, 6, 7], which is defined as persistent lumbar and lower extremity pain after lumbar spine surgery [1, 4]. In Europe, SCS is also approved for refractory angina pectoris and peripheral limb ischemia [3]. Spinal cord stimulator devices are comprised of a programmer, a pulse generator, an extension cable and electrode leads; leads can be percutaneous, paddle or hybrid leads [3]. A Tuohy needle is used to place percutaneous electrodes into the epidural space, while electrodes from paddle leads are placed surgically during a laminotomy or laminectomy [3]. Protocol for permanent placement includes a preliminary trial of stimulation where a patient is expected to report pain relief of 50% or more [3]. A radiograph of a post-surgical lumbar spine with implanted spinal cord stimulator can be visualized in Fig. 1.

### Outcomes

SCS has been found to significantly reduce pain, increase functional capacity, improve quality of life, reduce analgesic consumption, and allow patients to return to work [2, 6, 8–10]. The reported percentage of patients with chronic low back pain who experience 50% pain relief or more post SCS ranges from 50–62% [1, 8, 11], and reported patient satisfaction ranges from 70–85% [8, 9]. A systematic review and meta-analysis performed by Taylor et. al. reported 53% of patients no longer requiring analgesics and 40% of patients able to return to work post SCS; Taylor et. al. also reported a significant improvement in functional capacity and quality of life [8].

### Complications

Spinal cord stimulators are considered a relatively safe treatment option [3]. Reported rates of complications from spinal cord stimulator implant range between 18% and 43.5% [1–3, 6, 8, 9, 12, 13]. Life threatening complications are very rare [1], and neurological damage is uncommon [13]. The most commonly reported complication after implantation of a spinal cord stimulator is hardware malfunction, including electrode lead migration/breakage and implantable pulse generator migration; hardware malfunction occurs in 10-30% of patients [1, 3, 6, 9, 12, 13]. Biologic complications are significantly less frequent; infections occur in 3–5% of patients [1, 3, 6, 9, 12]. The majority of complications occur during the first 12 months after implant [12], and are usually minor and easily reversible with minor surgery [13].

### Predictors of Pain relief/Precautions SCS

In a 2014 systematic review and meta-regression analysis, Taylor et. al. found no significant diagnostic study, patient, or technology related predictors of pain relief following SCS [11]. Conversely, Deer et. al. concluded that indicators including the experience of the implanter, etiology of the patient’s pain, access to early treatment, the existence of comorbidities that might cause failure or electrode lead complications and a well performed psychologic evaluation can help determine the effectiveness of SCS [13]. Depressed mood, low energy levels, somatization, anxiety, and poor coping are predictors of worse outcome with SCS [13]. To minimize surgical complications, pre-surgery protocol calls for intravenous prophylactic antibiotics, and patients are advised to avoid extreme movements for the first six weeks after implantation to ensure the leads fix into place [9]. It is suggested that ongoing follow-up is needed to ensure optimal outcomes; Kumar et. al. recommend that a post-implant rehabilitation program to address deconditioning will assist patients in building functional capacity, returning to work, and actively participating in domestic and social life [6].

Despite the above mentioned methods of avoiding poor outcomes, only 50-60% of patients with spinal cord stimulators report 50% pain relief; 40–50% continue to experience pain [1]. Treatment options are limited for this patient population. In the absence of neurological deficit, conservative treatment options may be appropriate. One such option is chiropractic, specifically spinal manipulation.

### Manipulation

Spinal manipulation is performed by providing a high-velocity, low-amplitude (HVLA) thrust to a diarthrodial synovial joint. Often, manipulation is associated with an audible cavitation or cracking sound, which is caused by the formation and activity of bubbles within the synovial fluid when pressure is reduced within the joint [14]. There is moderate evidence for clinical efficacy of spinal manipulation therapy for acute and chronic low back pain [14–16], but the physiological mechanisms behind the effects of spinal manipulation are still unknown [14]; main theories include: release of entrapped synovial folds, relaxation of hypertonic muscles via sudden stretching, disruption of articular or periarticular adhesions, and hypoalgesia of the associated dorsal horn of the spinal segment manipulated [14].

Spinal manipulation is a relatively safe procedure; the most common adverse reaction being temporary local discomfort in 44–55% of patients [17, 18]. Serious adverse events are rare. Reversible side effects, such as progression of neurological deficits resulting from lumbar disc herniation are relatively uncommon, and irreversible complications are extremely rare; the risk of irreversible cauda equina syndrome is estimated to be associated with 1 in 100 million lumbar spine manipulations [15]. Specifically regarding spinal manipulation of the post-surgical spine, current evidence is limited to case studies and does not include any literature on spinal manipulation post spinal cord stimulator implantation [19].

In regards to chronic low back pain, approximately 6–7.5% of patients receive spinal surgery [20, 21]. Of these patients, up to 61% report experiencing lumbar pain after surgical intervention, [19, 22–24] and 2.3–12% of post-surgical patients receive chiropractic care [25–27]. Only case studies have been performed that address the safety and efficacy of chiropractic care of postsurgical spinal pain; positive outcomes with no major adverse effects were reported [19, 21, 28–35].

The main objective of this study is to describe the potential adverse effects of lumbar spine manipulation in post-surgical patients with spinal cord stimulators; the cases presented were drawn from patients presenting at the VA Connecticut Healthcare System.

## Case presentation

### Case #1

A 58 year-old male presented with a history of chronic low back pain and intermittent right, greater than left, lower extremity pain and numbness status post L3-4, L5-S1 hardware fusion and spinal cord stimulator implantation. His low back pain began approximately 24 years prior, after lifting a heavy object, and his bilateral lower extremity symptoms insidiously began several years later. Following fusion surgery in 2004, the patient was relatively pain free for approximately 7 years, after which his low back pain and left lower extremity pain reoccurred without an inciting event. It was at that time he underwent a successful spinal cord stimulator trial and subsequent implantation in 2011 which largely resolved his left lower extremity symptoms. However, shortly thereafter, he began to experience right lower extremity pain and numbness. At the time of presentation to our clinic, his low back pain was constant yet variable in intensity ranging from 3/10-8/10. It was provoked with sudden movements, standing, and lying down, and relief was achieved with sitting, moist heat, and opiates. He denied bilateral lower extremity weakness, saddle anesthesia, bowel or bladder dysfunction, unexpected weight change, fever, chills, nausea, vomiting, abdominal complaints, or temporal factors. Prior treatment had included acupuncture, physical therapy, and opiate and non-opiate analgesics which the patient found to be temporarily beneficial.

Past medical history was remarkable for hypertension, gastroesophageal reflux disease, obstructive sleep apnea, and right ulnar nerve entrapment. He had not used tobacco for many years, consumed alcohol rarely, exercised very little, and was employed as an electrical technician.

Examination revealed a well-nourished and well-groomed male in no apparent distress who was cooperative and pleasant. His gait and station were unremarkable, his speech was fluent, and he was alert and oriented to person, place, and time. A well-healed midline scar was noted over the lumbar spine secondary to his prior fusion surgery. Tibialis anterior and dorsalis pedis pulses were intact, strong, and symmetric bilaterally, without evidence of edema or palpable tenderness. Deep tendon reflexes (DTRs) were 1+ and symmetric with reinforcement at the patella and achilles bilaterally. Hypoesthesia was noted over the lateral right thigh and distal leg. Strength was 5/5 throughout the lower extremities bilaterally. Straight leg raise (SLR) and femoral nerve stress test were unremarkable for signs of nerve root tension. Lumbar spine range of motion was severely limited in flexion and mildly limited in extension with local low back pain in both directions. Repeated end range loading was unremarkable for centralization or peripheralization. Facet loading produced local low back pain bilaterally and bilateral sacroiliac provocation produced local non-concordant sacroiliac joint pain. Articular stiffness and pain was noted in the upper lumbar spine and bilateral sacroiliac joints, and hypertonicity and tenderness to palpation was noted throughout the lumbar paraspinal muscles and gluteal musculature. Hip provocation was unremarkable. X-ray of the lumbar spine demonstrated transpedicular screw fixation at L4-S1 with spinal cord stimulator placement over the left iliac crest and leads entering at the left T12-L1 level and migrating superiorly to the thoracic spine.

The patient was diagnosed with mechanical low back pain status post L3-4, L5-S1 fusion and spinal cord stimulator implantation. He was assessed for appropriateness of HVLA spinal manipulation by provocation testing involving the application of graded preloading consistent with the manipulative procedure. As there was no increase in his low back pain or provocation of lower extremity symptoms, he underwent a trial of treatment including HVLA spinal manipulation to the upper lumbar spine and lower thoracic spine, flexion-distraction mobilization to the lumbar spine, and myofascial release to the lumbar paraspinal muscles. He was treated 6 times over the next 3 months, reporting durable relief of his low back pain; he noted increased tolerance to standing and lying down (30 min vs. less than 5 min at the initial consultation) and reduction in pain levels to 2-5/10 depending on activity. No changes were noted in opiate usage. He reported experiencing no adverse reactions or onset of new symptoms post treatment.

### Case#2

A 57 year-old male presented with a history of acute-on-chronic low back pain and bilateral lower extremity numbness and tingling status post spinal cord stimulator implantation. He noted several years of low back pain and bilateral lower extremity numbness and tingling that was initially non-responsive to trials of physical therapy, chiropractic, aquatherapy, and lumbar epidural steroid injections. In 2013 he underwent spinal cord stimulator implantation after reporting greater than 60% reduction in low back pain and 95% reduction in his bilateral lower extremity numbness and tingling with a stimulator trial. He experienced approximately 2 years of durable relief post implantation until bending over to pick up a bar of soap; this resulted in a flare-up of his low back pain and bilateral lower extremity numbness and tingling. At the time of presentation to our clinic his symptoms had persisted for 3 weeks and remained unchanged despite trials of nonsteroidal anti-inflammatory drugs (NSAIDs), moist heat, and rest. His complaint was provoked with standing more than 10 min and walking. Mild relief was achieved with lying down and sitting. He denied bilateral lower extremity weakness, radiation, saddle anesthesia, bowel or bladder dysfunction, unexplained weight loss, fever, chills, nausea, vomiting, temporal factors, and change in symptoms with coughing, sneezing, or bearing down.

Past medical history was remarkable for depression, gastroesophageal reflux disease, type 2 diabetes mellitus, migraine headaches, morbid obesity, obstructive sleep apnea, and a remote history of polysubstance abuse. The patient was single, denied the use of alcohol or tobacco, and worked in customer service.

The examination revealed a mildly obese, well-groomed male who was cooperative and in no apparent distress. He was alert, awake, oriented to person, place, and time, his speech was intact and fluent, and his gait and station were within normal limits. A well healed scar was present midline in the lower lumbar spine. The patient’s DTRs were 2+ bilaterally and symmetric at the patella and achilles, strength was 5/5 throughout the lower extremities bilaterally and hypoesthesia was noted over the proximal anterior right thigh. SLR was negative both seated and supine, and femoral nerve stress test was unremarkable. Lumbar spine range of motion was full with mild end range low back pain during extension. Repeated end range loading was unremarkable for peripherlization or centralization. Facet loading was positive for concordant low back pain to the right, while sacroiliac and hip provocation were unremarkable. Articular stiffness and pain was noted in the lower lumbar spine and hypertonicity and tenderness was present in the adjacent lumbar paraspinal musculature. A CT of the lumbar spine demonstrated severe central spinal stenosis at L4-L5 and L5-S1 and a neurostimulator placed in the left superior gluteal region with lead tip entrance at L1-2.

The patient was diagnosed with symptomatic lumbar spine stenosis status post spinal cord stimulator implantation. He was deemed a candidate for side posture HVLA lumbar spine manipulation, as there was no increase in his low back pain, or provocation of lower extremity symptoms during pre-manipulative loading. He also received flexion-distraction mobilization to the lumbar spine and myofascial release to the lumbar paraspinal muscles. He was treated 5 times over the next 4 weeks reporting durable relief of his low back pain and bilateral lower extremity numbness and tingling to pre-injury levels. He also noted improved tolerance to walking and standing (30 min vs 10 min at the initial consultation). Moreover, he denied any adverse effect from treatment or onset of new symptoms post spinal manipulation.

### Case #3

An 81 year-old male presented with a history of chronic low back pain status post L4-5 laminectomy with fusion, T11-12 and T12-L1 laminectomy and fusion, epidurolysis x3, and spinal cord stimulator implantation. He initially noted low back pain and right lower extremity pain in the early 1980s that began insidiously and was non-responsive to conservative treatment measures. After the initial decompression and fusion in 1984, he reported moderate relief of both his low back pain and right lower extremity pain for several years prior to the return of symptoms and subsequent decompression and fusion in 2009. His symptoms returned again several years later; he then underwent epidurolysis in 2014 which did not result in any measurable benefit, per the patient. Eventually, due to the persistent nature of his complaint, a spinal cord stimulator trial was undertaken to which he responded positively. He subsequently underwent implantation in November 2014. He presented to our clinic noting chronic low back pain that was provoked with standing and walking, and relieved with sitting, bending over, lying down, opiates and with using a shopping cart while walking. He stated that his symptoms were worst in the morning. At the time of the consultation, the patient denied bilateral lower extremity weakness, radiating pain, numbness, or tingling, bowel or bladder dysfunction, saddle anesthesia, fever, chills, nausea, vomiting, unexpected weight change, or abdominal complaints. Prior treatment had included the above named interventional procedures, radio-frequency ablation x3, medial branch block, physical therapy, and opiate and non-opiate analgesics.

His past medical history was remarkable for coronary artery disease status post coronary artery bypass grafting, obstructive sleep apnea, benign prostatic hyperplasia, gastroesophageal reflux disease, and migraine headaches. Past surgical history included the above mentioned procedures in addition to bilateral cataract removal in 2000, bilateral carpal tunnel repair in 2001, bilateral total knee arthroplasty in 2007, right shoulder replacement in 2008, and a left rotator cuff repair in 2004. The patient resided with his wife, denied tobacco and alcohol use, and previously worked in manufacturing.

Evaluation demonstrated a well-nourished, well-groomed male who was cooperative, pleasant, and appeared in no apparent distress. His gait and station were unremarkable; he was alert, awake, oriented to person, place, and time with intact and fluent speech. Multiple well healed scars were present midline in the lumbar spine. DTRs were trace bilaterally and symmetric at the patella and achilles, strength was 5/5 and symmetric throughout the bilateral lower extremities, and sensation to light touch was intact bilaterally and symmetrically. Lumbar spine range of motion was moderately limited in all directions, however, he demonstrated a preference for lumbar spine flexion, as extension was painful. Articular stiffness and pain was noted throughout the lumbar spine with associated hypertonicity and palpable tenderness to the adjacent musculature. A CT of the lumbar spine demonstrated a T9-10 disc herniation without thecal sac encroachment, and a spinal cord stimulator with lead placement at T11-12.

The patient was diagnosed with failed back surgery syndrome status post spinal cord stimulator implantation. He was assessed for the appropriateness of HVLA spinal manipulation and underwent a trial of manual treatment consisting of spinal manipulation to the lumbar spine, flexion distraction mobilization to the lumbar spine, and instrument assisted soft tissue mobilization to the paralumbar musculature. He received 2 treatments and reported no benefit. However, he also reported no adverse reaction or onset of new symptoms post treatment. No further treatment was rendered as he was to undergo repeat medial branch blocks in the lumbar spine and wished to discontinue the chiropractic trial.

### Case #4

A 73 year-old male presented with a history of chronic low back pain and right lower extremity pain, weakness and, numbness status post L4/5 laminectomy and fusion, and spinal cord stimulator implantation. Prior to the initial surgery, the patient had an 18 year history of progressive low back and right lower extremity pain that began insidiously. During that time he had trialed and failed to respond to chiropractic, physical therapy, and acupuncture. Subsequently he underwent an L4/5 laminectomy and fusion in 2000. However, post-surgery, he noted progressive bilateral lower extremity weakness that mildly improved with a 2 year trial of physical therapy. Following that period, he reported continued low back pain and right lower extremity dysesthesia and pain which was subsequently treated with spinal cord stimulator implantation in 2010. He presented to our clinic 4 years post implantation with continued low back pain and right lower extremity pain that was provoked with walking more than ¼ mile, standing more than 10 min, golfing, and lifting heavy objects. Mild relief was achieved with NSAIDs, morphine, moist heat, and lying in a lateral decubitus position. He denied bowel or bladder retention or incontinence, saddle anesthesia, fever, chills, nausea, vomiting, unexpected weight change, change in symptoms with coughing, sneezing, or bearing down, or abdominal complaints. Prior treatment had included the aforementioned surgical procedures, physical therapy, repeat lumbar epidural steroid injections, and opiate and non-opiate analgesics.

Past medical history was remarkable for Type 2 diabetes mellitus, coronary artery disease, hypertension, hyperlipidemia, post traumatic stress disorder, major depressive disorder, benign prostate hyperplasia, resection of submandibular benign tumor, rotator cuff repair, and sensorineural hearing loss. The patient resided with his wife, had a remote history of tobacco use (greater than 40 years prior), and used alcohol socially. He was no longer working at the time of the encounter.

Examination revealed a well-nourished and well-groomed male in no apparent distress. His gait and station was unremarkable and he was alert, awake, oriented to person, place, and time, with intact and fluent speech. Multiple well healed scars were present midline in the lumbar spine. DTRs were 2+ brisk bilaterally and symmetric at the patella, and 2+ bilaterally and symmetric at the achilles. Strength was mildly decreased (4/5) globally in the bilateral lower extremities and hypoesthesia to light touch was noted over the right lateral lower extremity and right great toe. Adverse nerve root tension was noted with right SLR supine but not seated. Lumbar spine range of motion was moderately limited in all directions, without a directional preference. Articular stiffness and pain was noted throughout the lumbar spine with associated hypertonicity and palpable tenderness to the adjacent musculature. A CT scan of the lumbar spine demonstrated L4-S1 fusion hardware with posterior decompression and a neurostimulator placed posterior to the L2-3 spinous processes with lead tip entrance at L3-4.

The patient was diagnosed with failed back surgery syndrome and chronic right L4/5 radiculopathy status post L4/5 laminectomy and fusion, and spinal cord stimulator implantation. The patient could not tolerate pre-manipulation positioning thus HVLA spinal manipulation was not performed. Instead, he underwent a trial of care consisting of flexion distraction mobilization to the lumbar spine and myofascial release to the paralumbar musculature. The patient was treated 4 times over the next 4 weeks noting temporary relief of his low back pain and no change in his right lower extremity symptoms. He denied the presence of adverse reaction or post treatment soreness following each encounter. The trial was not continued as his response was not durable and he wished to re-engage with pain management for repeat interventional procedures.

## Discussion

6–7.5% of patients with low back pain receive spinal surgery [20, 21], and up to 61% of patients who receive lumbar surgical intervention report continued low back pain [19, 22–24]. Spinal cord stimulation has been used for over 40 years for the treatment of chronic pain [1–5], and is approved in the United States as an appropriate treatment for chronic trunk and limb pain, intractable low back pain, leg pain, and failed back surgery syndrome [3]. After implantation of spinal cord stimulators, 50-60% of patients report 50% pain relief [1]. In patients who continue to experience low back pain after implantation of a spinal cord stimulator, treatment options are limited. Conservative treatment options, including spinal manipulation, may be appropriate for this population if patients are not experiencing neurological deficit; 2.3–12% of post-surgical patients receive chiropractic care [25–27].

Patients with spinal cord stimulators are advised to avoid extreme movements for the first six weeks after implantation to ensure the leads fix in place [6]; there is currently no public data in regards to the physical forces required to cause lead fracture or dislocation, so we are unable to identify how these forces are related to the forces generated from spinal manipulative therapy. In an effort to minimize the opportunity for lead fracture, we limited physical contact to the patient’s spinal cord stimulator and took care to avoid excess torsional forces of the lumbar spine. At this time, there is no literature available on manual treatment or physical therapy for persistent pain status post stimulator implantation. In this study, we used knowledge of postsurgical spine biomechanics and examination findings to support the use of HVLA manipulation and/or mobilization as a treatment option for four low back pain patients with low back pain status post spinal cord stimulator implantation.

## Consent

Written informed consent to publish has been obtained from all persons involved in this study. This is an exempt study; IRB approval is waived.

## Conclusion

Four patients with chronic low back pain status post spinal cord stimulator implantation were treated with manual therapy; of these patients, two were treated with HVLA manipulation, and two were treated with lumbar flexion distraction mobilization. After treatment, two patients reported durable reduction in low back pain with increased tolerance to walking, standing, or lying down, one reported temporary relief of low back pain, and one reported no change in symptoms. All four patients denied adverse effects or onset of new symptoms after treatment. Our outcomes may have been affected by a higher incidence of mental health conditions in the veteran population [36]; some of these conditions have been shown to negatively impact outcomes in patients with spinal cord stimulators [13].

In multiple studies, spinal manipulation and/or mobilization has been shown to be a safe and effective treatment for the treatment of chronic low back pain [14–16]. Only cases studies have been performed that address the safety and efficacy of chiropractic care in post-surgical spinal pain [19, 21, 28–35], and no investigation has been done in regards to spinal manipulation as a treatment for chronic low back pain in patients with implanted spinal cord stimulators. In patients with continued low back pain after implantation of a spinal cord stimulator, where further spinal surgeries or pharmacological treatment are not indicated, spinal manipulation and/or mobilization may be considered. This is Level 4 evidence (case study) and as such one cannot use it to conclude efficacy; it is important to note, however, that this study has demonstrated the absence of adverse effects from manipulative or mobilization treatment in patients with spinal cord stimulators. Further investigation is needed to determine the appropriateness of spinal manipulation in patients with implanted spinal cord stimulators.

## Abbreviations

SCS: Spinal cord stimulation
FBSS: Failed back surgery syndrome
HVLA: High-velocity, low-amplitude
DTR: Deep tendon reflex
SLR: Straight leg raise
NSAID: Nonsteroidal anti-inflammatory drug
